# Professional Development to Inspire, Support, and Extend STEM-Related Learning

**DOI:** 10.3390/bs16010127

**Published:** 2026-01-16

**Authors:** Somayeh Ba Akhlagh, Asma Hulayyil Aljohani, Maryam Jamal Alharthi, Nahla Mahmoud Gahwaji, Nouf Mohammed Albadi, Marianne Knaus

**Affiliations:** 1 Faculty of Humanities, Arts, Social Sciences and Education, School of Education, University of New England, Armidale, NSW 2350, Australia; somayeh.baakhlagh@une.edu.au; 2 Department of Educational Psychology, Faculty of Education, Taibah University, Madinah P.O. Box 41411, Saudi Arabia; azaeide@taibahu.edu.sa (A.H.A.); mjharthy@taibahu.edu.sa (M.J.A.); 3 Department of Early Childhood, Faculty of Human Sciences and Design, King Abdulaziz University, Jeddah 80204, Saudi Arabia; ngahwaji@kau.edu.sa; 4 Faculty of Education Departments of Curriculum and Teaching Methods, King Abdulaziz University, Jeddah 80204, Saudi Arabia; nmalbadi@kau.edu.sa; 5 School of Education, Edith Cowan University, Perth, WA 6027, Australia

**Keywords:** STEM, early childhood, professional development, Vygotsky, teacher, cultural context

## Abstract

The success of STEM education in early childhood education is reliant on the pedagogical practices of teachers. Effective teaching of STEM requires specific knowledge of the four disciplines of STEM, appropriate teaching and learning methods and relevant experiences. In Saudi Arabia the teaching of STEM is a relatively new field, and this paper outlines a research project to promote the teaching and learning of STEM through professional development workshops. The research is informed by Vygotsky’s cultural-historical/socio-cultural theory, acknowledging the crucial role of social interaction and cultural context in a collaborative learning environment. To evaluate the project, a mixed methods approach was used involving the collecting, analyzing, and interpreting of quantitative and qualitative data. Surveys were conducted before and after professional development as well as semi-structured interviews. The findings indicate positive shifts in attitudes and enthusiasm among early childhood educators to teach STEM following the professional development program. However, the practical implementation remains a challenge due to the perceived lack of suitable resources, support from school leadership and the need for ongoing coaching and mentoring.

## 1. Introduction

STEM is described as an integrated instructional approach that brings together science and mathematics through scientific inquiry, technological and engineering design, and mathematical analysis (Johnson, 2013; Zendler et al., 2018), while recognising that children’s interest in STEM-related learning can be effectively leveraged through play-based experience (Wan et al., 2020; Knaus, 2023). The teaching of STEM in early childhood is increasingly relevant in the current educational context as it lays the foundation for essential skills that influence both children’s everyday lives and their future opportunities (Ghazali et al., 2024). Teachers incorporate these four disciplines within their teaching, fostering integrated learning experiences across the STEM spectrum (Australian Education Department, 2021). Globally, many countries have prioritized STEM through their educational policies, recognizing its role in preparing future generations for the demands of a rapidly evolving world (UNESCO, 2019). Early childhood education must be viewed as a pivotal stage for STEM learning as it is widely acknowledged as a critical period of life in which early experiences profoundly shape lifelong learning and development (Sylva et al., 2010; World Health Organization, 2020). Moreover, young children are naturally inclined to explore, question, and experiment, qualities that make them natural scientists and engineers (Chesloff, 2013). They also possess an exceptional capacity for learning during the early years (Victoria Department of Education and Training, 2017), making this an ideal time to foster critical thinking, problem-solving (Akcay Malcok & Ceylan, 2022) and scientific inquiry through STEM-based experiences (Tytler, 2020). These skills not only enrich immediate learning but also support educational attainment and career development in a future where STEM-related competencies are increasingly valued (Cohrssen & Garvis, 2022).

However, despite its well-recognised importance, STEM applicability poses several challenges. Bagiati and Evangelou (2015) and Haney et al. (2002), state that teachers are the most important contributors to children’s STEM-related learning. Yet, Alghamdi’s (2022) research found that teachers lacked appropriate knowledge and understanding of STEM education in early childhood settings. Unfamiliarity with STEM pedagogy may cause teachers to not prefer applying this approach in everyday teaching. Park et al. (2017) in their study also reasoned that school administrators prioritized students’ reading, writing, and other mathematics skills over STEM opportunities. Pedagogical practices in STEM teaching may vary depending on teachers’ knowledge, access to resources, available time, and confidence (Aldemir & Kermani, 2017; Alexander et al., 2014; Ong et al., 2016; Park et al., 2017; MacDonald et al., 2021). Despite variations in teaching approaches, STEM pedagogical practices may include integrated approaches to teaching, play-based learning, and inquiry learning (Knaus, 2023). Alghamdi (2022) considered whether teachers lack knowledge on methods and abilities on how to integrate the four disciplines of STEM was another contributing factor to the absence of implementation.

One way to support teachers to implement and integrate STEM pedagogical practices is through targeted Professional Development (PD) to enhance their knowledge and confidence. PD is also referred to as professional learning and can be applied through various approaches including mentoring, professional meetings, attending workshops, online learning, reading, or watching documentaries (Boz, 2023). Research has shown that participation in PD programs can significantly improve early childhood teachers’ ability to plan and implement STEM experiences effectively (Davila Dos Santos et al., 2021; DeJarnette, 2018). Walshe et al. (2025) proposes that without appropriate teaching and guidance it is not likely teachers will include STEM in their pedagogical practices. Purposeful planned PD is essential for equipping teachers with the knowledge and skills needed to enhance instructional quality and improve children’s learning outcomes (Copur-Gencturk & Thacker, 2020). According to Slavit et al. (2016), teachers’ positive attitudes towards STEM, their teaching methods, practices, and instructions can significantly affect their learners’ perceptions and engagements in STEM learning. Walshe et al. (2025) suggests that a teacher’s willingness and ability are two factors that will influence their ability to effectively implement STEM experiences. One effective approach to PD is to model STEM experiences and for teachers to observe how to plan opportunities to incorporate the four STEM disciplines in their planning and practice (Darling-Hammond et al., 2017). According to research, using a modelling approach encourages active learning which is more effective in achieving targeted knowledge and skills (Bates & Morgan, 2018; Penuel et al., 2007). Furthermore, Boice et al. (2021) endorses the use of hands-on activities to demonstrate to teachers how to integrate STEM learning. The collaborative features of PD highlight active learning and thus ensure better learning outcomes (Darling-Hammond et al., 2017). Boz (2023) recommends PD as an important part of teacher development that is not only a government or administrative approach to learning, but a popular practice preferred by teachers.

Studies from Australia (MacDonald et al., 2021) and Saudi Arabia (Ba Akhlagh & Allehyani, 2023) have reported that early childhood teachers often lack confidence in teaching STEM. This deficit possesses a barrier to the implementation of STEM education. A study in Saudi Arabia (Madani, 2020) indicated a large number of teachers were unable to give a clear description of what STEM education stands for, reflecting the teachers’ lack of readiness and self-confidence to enact STEM integrated practices efficiently. Seemingly, there exists a gap between the available professional development programs that address short-term issues and what is truly needed to guarantee a transformational reform in STEM teaching practices in Saudi Arabia. Ba Akhlagh and Allehyani (2023) also confirm a lack in existing literature in Saudi Arabia on STEM education for early childhood. In Saudi Arabia, STEM learning is shaped by the sociocultural context in which teachers work. The Saudi education system is deeply influenced by the Kingdom’s social structure, cultural traditions, and Islamic values, which shape educational policies, curriculum design, and teaching practices (Almalki, 2023). These social, cultural, and religious foundations emphasize collective responsibility, moral development, and social cohesion, influencing how teachers engage in professional learning and enact STEM reforms in early childhood contexts (Alghamdi, 2023). For example, cultural values emphasizing community involvement and the country’s educational policies and systems all play a significant role in influencing teacher learning (Vygotsky, 1978). In the Saudi Arabia strategic plan referred to as Vision 2030 (2016) it outlines plans for the future regarding social and economic reforms with long term goals and expectations for the Kingdom. Within the plan, there is an emphasis on promoting the economy by providing opportunities within the workforce for everyone through life-long training “so they may contribute to the best of their abilities” (Vision 2030, 2016, p. 37).

STEM education can act as a driver for change and development in many sectors, including economics, technology, and, more importantly, education (Mohamad Hasim et al., 2022). Accordingly, many governments and educational institutions all over the world are prioritising STEM learning as a planned investment to ensure national development and economic sustainability. The Saudi Arabia Vision 2030 proposes; “We will invest particularly in developing early childhood education, refining our national curriculum and training our teachers and educational leaders. We will also redouble efforts to ensure that the outcomes of our education system are in line with market needs” (Vision 2030, 2016, p. 36). The current research aims to investigate how professional development (PD) programs can inspire, support, and extend teachers’ abilities to provide high-quality STEM practices in early childhood education in Saudi Arabia. Targeted professional development has the ability to empower teachers, enhance instructional quality, and may also result in long-term gains in STEM learning outcomes. Darling-Hammond et al. (2017) and Ba Akhlagh and Allehyani (2023), for instance, claimed that effective PD is a fundamental element that improves instructional methods and enhances teachers’ confidence when applying STEM practices.

In line with the above discussion, this study mainly addresses the following objectives:

1. To analyse the impact of a targeted professional development on STEM education for early childhood teachers within the Saudi Arabian education context.
2. To identify how early childhood teachers apply their knowledge derived from the professional development program into practice when teaching STEM within the Saudi Arabian education context.

To focus on these objectives this study included 10 (5 in Jeddah and 5 in Madinah) PD workshops in two major city locations in Saudi Arabia. The workshops addressed what is STEM, why it is relevant in early childhood, the pedagogical practices of play-based learning of STEM, relevant theories and hands on practical experiences that were modeled during the session. Hands-on practical experiences included ten examples: building a car ramp; constructing a tower; creating a stop-motion animation video; playing the Robot Turtles board game; building a bridge (beginning with reading The Three Billy Goats Gruff, followed by designing and constructing a bridge using a variety of materials); using Bee-Bots; coding a LEGO maze; exploring sinking and floating (accompanied by the story Who Sank the Boat?); measurement (linked to the story How Big Is a Dinosaur?); and volume and floating (accompanied by the story Alexander’s Outing). In addition, we provided different scenarios demonstrating how teachers can support children’s STEM learning. A website was developed (www.stem4all.education) that included further resources, readings and practical experiences for the participants to refer to after the workshops to apply to their teaching practices. The PD provided a supportive communal learning environment as recommended by Boz (2023) and helped teachers develop confidence in implementing STEM within the curriculum.

The research data collected from the participants provided valuable information to support teachers in their role of implementing STEM experiences and to inform further PD opportunities.

## 2. Methodology

This study adopted a sequential explanatory mixed-methods design (QUAN → QUAL), where the quantitative phase was prioritized and used as a primary component, followed by a qualitative phase designed for clarification (Creswell & Clark, 2017). The quantitative phase involved a structured survey examining patterns, relationships, and group differences among key variables. Integration occurred at multiple stages of the research process. Survey results informed participant selection for the qualitative phase using criterion-based and maximum variation sampling (Patton, 2015), based on quantitative response profiles (e.g., high, medium, low, and unexpected patterns). Quantitative findings also directly shaped the interview protocol, with questions targeting statistically meaningful trends, subgroup differences, and ambiguous results. At the interpretive level, integration was achieved through a coherent narrative in which qualitative themes were used to explain and contextualise quantitative findings, producing integrated meta-inferences and enhancing methodological rigour in line with mixed-methods standards (Fetters et al., 2013).

The research was grounded in Vygotsky’s (1978) socio-cultural theory, which emphasizes learning as a socially mediated process. This theoretical lens was appropriate because the professional development workshops were designed to encourage collaborative reflection and cultural responsiveness in STEM teaching within early childhood contexts. The study involved two groups of participants from Jeddah and Madinah, Saudi Arabia. The quantitative phase included 77 early childhood teachers, with roughly equal representation from both cities, who took part in pre- and post-workshop surveys. All participants were female teachers working in early childhood settings and attending professional development workshops focused on STEM education. Participants had diverse educational backgrounds, including diplomas, bachelor’s degrees, and postgraduate degrees in early childhood education or related fields. The qualitative phase featured semi-structured interviews with 16 participants chosen purposefully to reflect diversity in educational background, teaching experience, school type (public and private), and levels of engagement with STEM education. Participants’ teaching experience ranged from early-career to highly experienced educators. Additional details about the educational qualifications, years of experience, and institutional contexts of the interview participants are shown in Table 1.

### 2.1. Quantitative Phase (Survey)

Two structured surveys were administered one before and one after the professional development workshops to measure changes in teachers’ confidence, attitudes, and readiness to integrate STEM. The survey items were adapted from established instruments in STEM education research (Aldemir & Kermani, 2017; Thibaut et al., 2018) and contextualised for Saudi educational settings. Responses were collected using a five-point Likert scale ranging from “strongly disagree” to “strongly agree.”

### 2.2. Qualitative Phase (Interviews)

Following the survey analysis, semi-structured interviews were conducted with 16 volunteer participants to explore their reflections on the workshops and their experiences implementing STEM approaches in their classrooms. The interviews were guided by open-ended questions that encouraged discussion of challenges, perceived benefits, and contextual influences on STEM teaching. Each interview lasted between 40 and 60 min and was conducted in Arabic, then transcribed and translated into English for analysis.

### 2.3. Data Analysis

Survey data were analysed using the Statistical Package for the Social Sciences (IBM Corp, 2022). Descriptive statistics and paired-sample t-tests were employed to identify significant differences between pre- and post-workshop scores. This analysis provided an overview of shifts in participants’ perceptions and confidence in teaching STEM (Field, 2018). Interview data were analysed using reflexive thematic analysis following Braun and Clarke’s (2021) six-phase framework. All interview transcripts were imported into MAXQDA to support systematic coding, data organisation, and auditability. Initial open coding was conducted inductively at the semantic level to capture teachers’ expressed meanings, classroom practices, and reflections on professional development. The coding process was undertaken independently by the second and third researchers, both of whom have expertise in early childhood education and qualitative research methods. First-cycle codes were iteratively reviewed, compared, and refined through analytic discussion, leading to the development of a shared coding framework. Related codes were then clustered into higher-order categories, resulting in three overarching themes: (1) Professional Development as a Catalyst for Pedagogical Change, (2) Strengthening Teachers’ Capacity for STEM Implementation, and (3) Holistic Integration of STEM through Play-Based Learning, each comprising multiple analytically distinct sub-themes.

Consistent with a reflexive thematic analysis approach, formal interrater reliability statistics were not calculated. Instead, analytic rigor was ensured through iterative consensus-building between the two coders, transparent documentation of coding decisions within MAXQDA, and continual comparison across cases to confirm thematic coherence. Thematic interpretation was guided by socio-cultural theory to link teachers’ reflections with the collaborative and contextual nature of professional learning.

### 2.4. Ethical Considerations

Ethical approval for this study was granted by the University of New England (Australia) ethics committee. Participants were provided with detailed information sheets and signed informed consent forms prior to participation. Confidentiality was maintained by assigning pseudonyms, and all data were stored securely in accordance with the ethical requirements of both institutions.

### 2.5. Rationale for Chosen Methods

The explanatory sequential mixed-methods design was chosen to capture both the measurable outcomes and the lived experiences of teachers participating in professional development. Quantitative surveys allowed for the identification of general trends, while qualitative interviews provided insight into the contextual factors underlying those trends. This integration of data types strengthened the interpretation of results, enabling a holistic evaluation of the professional development program’s impact on STEM teaching in early childhood education in Saudi.

## 3. Results

### 3.1. Quantitative Data

#### Validity and Reliability Results of the Survey

To verify the internal consistency validity of the survey, the correlation coefficient between the scores of each question in the survey and the total scores of the survey was calculated. The results are shown in Table 2.

Table 2 shows the correlation coefficients between the scores for each question in the survey and the total scores for the survey, ranging between (0.75–0.83) and all statistically significant. Thus, the survey questions are considered valid for what they were designed to measure. To verify the reliability of the survey, Cronbach’s alpha method was used, and the results are shown in Table 3.

Table 3 shows the stability coefficient for the survey, which was (0.81), which is an acceptable ratio. A paired samples t-test was used to identify if there was a statistically significant difference between the average scores of the research sample’s responses to “I need training to teach STEM to children” before and after attending the workshop. The results were as follows:

Table 4 shows that there is a statistically significant difference between the average scores of the research sample’s responses to “I need training to teach STEM to the children” before and after attending the workshop, where the average score before attending the workshop was (3.14), which is at the “average” level, while the average score after attending the workshop was 4.51, which is at the “high” level. The t-value was 8.64 and the significance level was 0.001.

To examine if there was a statistically significant difference between the average scores of the research sample’s responses to “I confidently incorporate STEM programs based on children’s interests” before and after attending the workshop, a paired samples t-test was used.

There is a statistically significant difference between the average scores of the research sample’s responses to (I confidently incorporate STEM programs based on children’s interests) before and after attending the workshop, where the average score before attending the workshop was (2.58), which is considered “low,” while the average score after attending the workshop was (4.21), which is at the “high” level. The t-value was (8.96) and the significance level was (0.001).

A paired samples t-test was used, to identify if there was a statistically significant difference between the average responses of the research sample to the statement ‘I think STEM education is important equally for boys and girls’ before and after attending the workshop?”.

There is a statistically significant difference between the average scores of the research sample’s responses to (I think STEM education is important equally for boys and girls) before and after attending the workshop, where the average score before attending the workshop was (3.18), which is at the “average” level, while the average score after attending the workshop was (4.55), which is at the “high” level. The t-value was (7.78) and the significance level was (0.001).

A paired samples t-test was used to identify if there was a statistically significant difference between the average scores of the research sample’s responses to (Boys and girls equally benefit from STEM education) before and after attending the workshop.

There is a statistically significant difference between the average scores of the research sample’s responses to the statement “Boys and girls equally benefit from STEM education” before and after attending the workshop. The average score before attending the workshop was (3.26), which is considered “average,” while the average score after attending the workshop was 4.68, which is at the “high” level. The t-value was 12.05 and the significance level was 0.001.

When considering if the targeted professional development program had an impact on early childhood teachers’ knowledge of STEM and their self-confidence a paired samples t-test was used to indicate the differences between the mean total scores of the survey before and after attending the workshop. The eta-square equation (n2) was used to calculate the magnitude of the proposed program’s impact. Cohen provided an explanation of the value of “effect size,” which is small if the value of the square of the eta is 0.01, medium if the value is 0.06, and large if the value is 0.14. There is a statistically significant difference between the average scores of the research sample’s responses on the Overall Evaluation before and after attending the workshop, where the average score before attending the workshop was (3.04), which is at the “average” level, while the average score after attending the workshop was 4.48, which is at the “high” level. The t-value was 15.46 and the significance level was 0.001 and the impact of the professional development program was 0.76, indicating that the professional development program used by the researcher had a significant impact and led to an increase in early childhood teachers’ knowledge of STEM fields and their self-confidence.

## 4. Findings and Discussion of Qualitative Data

The qualitative interview analysis identified four interrelated themes that capture teachers’ experiences of translating STEM professional development into early childhood practice. These themes reflect variations in how workshop knowledge was enacted in classrooms, the holistic integration of STEM through play-based learning, the visibility of STEM through community links, equity, and engagement, as well as the challenges encountered when implementing STEM professional development in practice. Table 5 synthesises these themes and presents representative participant excerpts to illustrate the analytic patterns identified across the dataset. The table provides a structured overview of the qualitative findings and complements the detailed narrative analysis presented in the following sections.

### 4.1. Theme 1: Applying Workshop Knowledge in Practice

Teacher interviews revealed valuable insights into how they effectively transferred workshop concepts into their classroom practices, enriching literacy, storytelling activity and numeracy experiences. One teacher explained how she implemented the workshop approach during story time by deliberately structuring the session and making the STEM links explicit:

*First, I choose a story that children like. I introduce the author and narrator and talk about the purpose of the front and back covers. Then I reread with attention to key vocabulary antonyms and synonyms and we discuss the sequence of events. For example, with a story about Rami and making wool, I integrated STEM: where wool comes from is science; the steps in producing wool involve mathematics (ordering and measuring); the different colors and forms suggest engineering (designing and combining shapes); and the machine we use represents technology.*

*(Areej)* 

Areej’s account illustrates applied PD knowledge in concrete domain mapping. She translates a single narrative of making wool into science (origins/properties of materials), mathematics (ordering processes; measuring length/quantities), engineering (design choices; combining shapes/fibres), and technology (spinning/loom machines). This makes each domain visible and nameable to children. Keene and Garvis (2024) suggest the implementation of an integrated approach represents a real-life situation demonstrating an interdisciplinary approach to STEM learning.

A second teacher extended the same logic by showing how representation tasks at the end of the literacy cycle consolidate both math reasoning ordering and scientific discourse explaining why events unfold:

*We draw the events of a story on white paper using colors or pencils. Each child numbers their drawing to show what happened at the beginning of the story (chicken and her children), then in the middle what the chicken did when her child felt down, then at the end the chicken protected her children and back them home… children were draw the characters of the chicken and color them. While they work, I talk with them, so they explained more about chicken, numbering a story ordered as well all these are linked to STEM.*

*(Nada)* 

By asking children to number their panels, Nada operationalizes early mathematics through ordering, one-to-one correspondence, and before and after concepts in the story of the chicken. As learners explain what happened and why, she invites scientific reasoning grounded in causality and evidence drawn from the text and images. The drawing of scenes and characters adds an engineering and design dimension, requiring planning and the composition of parts into a coherent whole while sustaining attention and engagement, facets which according to Ritoša et al. (2023) are central priorities in early childhood education. Importantly, all of this occurs within the existing literacy block, indicating that the PD ideas have been absorbed into daily structures rather than added as extra workload.

In addition, teachers leveraged common tools (e.g., cubes) across engineering, computing, mathematics, and even language, reinforcing that STEM is not siloed but interwoven with literacy. Other teachers emphasized the adaptability of tools across subject areas. As one participant noted:

*Children can use cubes in engineering, building with them, but they also use them in computing and mathematics. Even in Arabic language activities, they form letters and numbers with the cubes. They used them for counting and for creating different shapes across many domains. Not only this, but they also asked if they could make more complex shapes, such as pentagons and hexagons.*

*(Anfal)* 

This example points out that children’s growing mathematical curiosity and higher-order thinking are evident as they move beyond simple shapes into more complex geometric forms. This meant that after the workshop, teachers began to view STEM not as isolated subjects but as interconnected, which aligns with play-based pedagogy emphasized in local research and policy (Keene & Garvis, 2024; Cohrssen & Garvis, 2022). Moreover, Tahani provided another demonstration and explained how her colleagues in the same class presented a literacy lesson with rich information, including an example of how she explained holograms and the five senses in the human body. She said:

*My colleague taught a lesson about the five senses, and this is how she used the app. First, she showed a hologram, which is a reflected 3D image. She linked it to science by dividing the class into five groups, one for each sense. For example, the group focusing on sight watched a video about the eye and its parts, and each group watched a video related to its assigned sense through the hologram. The classroom was very dark, and the children could only see the hologram reflection through the glass device she had set up. The children watched the display, asked questions, and we answered them. Then we started asking: ‘What did we see? Which sense did your group focus on?’ After that, we talked about the benefits of the senses and how many they are. She also used this to highlight Allah’s power in creating colors and shapes, how Allah created each organ, its form and size. She included counting as well how many ears, how many fingers so the children used numbers. In this way, we integrated STEM throughout the entire lesson.*

*(Tahani)* 

Tahani’s explanation demonstrates an innovative, cross-disciplinary experience that integrates STEM, digital technology, and religious values. By using a hologram to explore the five senses, the lesson engaged students in active learning, encouraging inquiry and hands-on discovery. Additionally, the teacher linked scientific concepts to the beauty of Allah’s creation, reinforcing both scientific understanding and spiritual reflection through play and play-based learning. Alharthi and Aljohani (2025) argue that play-based learning represents the primary pedagogical approach implemented in early childhood settings in Saudi Arabia. This perspective highlighted how STEM could foster both cognitive skills and imaginative expression in literacy and numeracy which make learning more engaging and meaningful for children. Teachers used technology as a scaffold to structure steps, prompt counting, and encourage repetition, while keeping inquiry playful and hands-on.

### 4.2. Theme 2: Holistic Integration of STEM in Play-Based Learning

Most teachers felt able to adapt STEM content to children’s natural play and curiosity, a central principle in early childhood pedagogy (Chesloff, 2013). The findings suggest that the workshops not only enhanced subject knowledge but also fostered a more integrated approach, in which mathematics, science, and technology were woven into everyday play activities and discovery learning (Knaus, 2023). The PD translated into concrete routines where children tried, recorded, and reflected within discovery corners and everyday classroom activity. Reem redesigned her classroom activities to integrate numeracy lessons into play-based corners. Reem explained:

*We might use the STEM concept without even knowing the term… Perhaps after the workshop, I learned more. For example, in the number lesson, I started integrating numbers into the STEM corners. In the discovery corner, I might include a butterfly or bees life cycle, already numbered and in different shapes. This way, children learn about numbers, math, shapes, geometry, the butterfly life cycle, and science. I also introduce a microscope, which represents technology. Now, I incorporate numbers and STEM ideas into every corner.*

*(Reem)* 

Reem’s approaches highlight how the workshop empowered them to connect a numeracy lesson with broader STEM concepts, demonstrating an essential teaching technique according to Knaus (2023). Reem’s integration of STEM into different learning corners reflects how educators can seamlessly combine numeracy with scientific exploration, fostering a holistic learning environment (Stone, 2024).

Teachers also emphasized that holistic integration of STEM through play-based learning nurtured children’s self-confidence and encouraged them to build positive attitudes toward STEM from the very beginning. One teacher explained that:

*STEM in playful, interest-driven activities helped even the youngest learners develop confidence and curiosity, supporting them to see STEM not as something distant or abstract but as part of their daily experiences.*

*(Abeer)* 

Abeer reflected on how the outcomes of the PD translated into classroom practices that fostered children’s motivation and supported their identities as confident STEM learners. Merry (another participant in the study) supported Abeer’s perspective by providing the interviewer an example, she described using a discovery corner to connect science and engineering through purposeful activities:

*I can use a screen so the child can see the required steps and count them. For example, building a beehive helps them understand how bees lay eggs, the wonders of God’s creation, and then they can try it themselves, repeat the steps, and even draw the experiment afterwards. In the discovery area, children record their experiment by drawing, which develops both creativity and engineering skills.*

*(Merry)* 

This reflection illustrates how the workshop equipped teachers with practical strategies to embed STEM in what Roberts and Knaus (2018) refer to as meaningful, play-based learning. Such approaches highlight how teachers translated workshop ideas into activities that balanced scientific inquiry with creativity (Stone, 2024), reinforcing the value of PD. Another teacher highlighted how technology and interactive resources attracted participation across the classroom:

*In engineering it’s not advanced engineering but using math-related activities with tools. I noticed that when I bring electronic or technological materials, nearly all the children want to participate—both boys and girls.*

*(Oadian)* 

This demonstrates how digital and technological stimuli were used to increase motivation and broaden engagement considered by Ji et al. (2024) as an important addition to STEM teaching. Oadian’s attitude reflects the values of innovation and sees technology as a bridge to connect abstract concepts like mathematics with hands-on, enjoyable experiences (Roberts & Knaus, 2018). In practice, technology acted as a bridge between children’s spontaneous play, play-based learning and structured STEM reasoning: it cued steps, amplified participation, and supported documentation, thereby embedding STEM across daily activities and reinforcing the theme of integrated learning.

Artistic practices (drawing, coloring, design) became vehicles for engineering design thinking and scientific representation, showing progression from simple coloring to purposeful design over the year. PD encouraged teachers to treat creativity as central to STEM learning, an aspect often overlooked according to Stone (2024). One teacher reflected:

*I noticed so much creativity among the girls in drawing, coloring, and design. At the beginning of the year they were only using colors, but later they developed to create designs such as sand. For example, draw a plant and its roots, soil, clouds, sun, roses and encourage them to draw more… These drawings are related to the STEM concepts in the different shapes of the sun and clouds are engineering, in plants and their roots they are science, and so on.*

*(Banan)* 

Banan’s example emphasizes how the PD encouraged teachers to view creativity not just as an add-on, but as a central component (Stone, 2024) of STEM learning, bridging artistic and scientific domains. Abeer also highlighted the benefit of the workshop in thinking differently and providing children with materials to support their creativity. She indicated that:

*After the STEM workshop I attended with you, I began to think more carefully about how to support children. I provided a math geometry kit with different shapes in the art corner. All the children’s girls and boys… loved it. they became creative, drawing houses in different sizes, varied colors, and beautiful shapes.*

*(Abeer)* 

This account highlights how PD encouraged teachers to nurture creativity as a vital part of STEM education, linking scientific, mathematical and engineering concepts with artistic expression. These accounts indicate that teachers moved beyond subject-specific teaching to embrace a holistic model of STEM, in which children’s interests, creativity, and play were central (Walshe et al., 2025). Nadiyah’s account highlights how the STEM workshop acted as a catalyst for teacher as well as child creativity. She explicitly notes that:

*To be honest, the STEM workshop allowed me to think more carefully before planning any activities for children. I started trying to be more creative in preparing my lessons, not like before. For example, I thought about explaining a lesson through an Expo theme. An Expo was coming up on a week before, so I sent a letter to the parents informing them that there would be an Expo. I presented a project, and my class created a design representing the Expo. My idea was to use environmental materials to create anything related to the Expo, from images and tools to innovations. I started thinking about how to use these tools and ideas in ways that could benefit the community.*

*(Nadiyah)* 

Nadiyah suggests a shift in her own professional identity from a deliverer of lessons to a creative designer of learning experiences. The workshop seems to have encouraged more intentional planning, and the development of reflective practice, elements highlighted by Veziroglu-Celik et al. (2025) as necessary for deep and thoughtful planning of STEM. Instead of relying on routine methods, Nadiyah pauses to plan, imagine alternatives, and connect activities to broader purposes through a STEM-rich, play-based learning opportunity. PD provided the knowledge, tools, and confidence to integrate mathematics, science, technology, and engineering across multiple domains of classroom activity. These findings align with recent research emphasizing that STEM in early childhood is most effective when embedded in purposeful play, supporting both cognitive development and creative problem-solving (Revák et al., 2024; OECD, 2022).

### 4.3. Theme 3: Making STEM Visible: Community Links, Equity and Engagement

Following the workshops, teachers reported concrete shifts in practice that widened participation and sustained curiosity beyond the classroom. They described using engaging starters and individualized supports more deliberately planned to connect children with STEM ideas through questions, movement, songs, and attractive tools. As Sara noted:

*Whether as a mother or a teacher, there is no other way than to bring attractive tools and start with an engaging introduction… involving children and asking pre-questions increases their enthusiasm.*

*(Sara)* 

These strategies, strengthened by the PD, linked engagement with STEM readiness and motivation. Teachers also felt better prepared to respond to diverse behavioral and emotional needs:

*I once had a boy with very high abilities in math and engineering… I had to first understand his family context and behavior before I could support him.*

*(Merry)* 

The workshop’s emphasis on responsive, child-centered pedagogy translated into more adaptive supports. The workshops also catalyzed outward-facing practices that embedded STEM in community life. As a direct outcome of the workshop, teachers-initiated community-linked experiences that made STEM visible beyond the classroom.

*We [as teachers] could plan science fairs and interactive in our community that connect directly to STEM concepts, allowing children to explore and learn in creative and meaningful ways.*

*(Aish)* 

Building on this, the workshop enabled teachers to *implement* those ideas by planning locally run STEM experiences workshops, school science fairs, and hands-on sessions with community partners, so classroom inquiry flowed into real-world practice. STEM research that includes families and communities is extremely underrepresented (Deehan et al., 2025) yet the inclusion of parents and wider cultural connections is relevant to bridge the home/school contexts and to be beneficial for all involved.

Teachers saw their classroom practices as embedded in wider cultural and national reforms, connecting PD impact to systemic shifts. At this point, Abrar provided an example of another dimension of collaboration, describing how children’s STEM-related learning was reinforced through partnerships with cultural and educational institutions. She suggested:

*To create a special ‘Science Day’ that would showcase simple experiments for children, making STEM learning visible and engaging [in addition] “There was a joint exhibition for children’s artwork with the school library and King Fahd Library, where children’s stories linked to STEM concepts were displayed.*

*(Abrar)* 

This example illustrates how PD outcomes inspired broader community engagement, situating children’s play-based STEM learning within local cultural spaces and strengthening the integration of STEM into children’s everyday environments. This aligns with Alharthi and Aljohani’s (2025) study, where preschool teachers in Saudi Arabia highlighted the importance of implementing play-based learning to enhance children’s learning and development, as well as integrating STEM concepts into their everyday environments (Alghamdi, 2022). Together, these accounts indicate that the PD enhanced teachers’ ability to use collaborative, interactive, and adaptive strategies, while also reinforcing awareness of how national policy changes shaped their pedagogical readiness. These findings are consistent with research showing that PD is most impactful when it equips teachers to adapt to diverse learners, promote inclusive collaboration, and situate classroom practice within broader socio-cultural contexts (Darling-Hammond et al., 2017; OECD, 2022).

### 4.4. Theme 4: Challenges in Translating the STEM PD into Practice

Interviews gave depth to these findings by illustrating how such challenges were experienced in daily practice. Several teachers emphasized that large class sizes and time constraints were among the most pressing barriers. As one explained:

*The number of children is very large, and the time is limited. You can barely spend time with each child to understand their needs and interests… By the end of the day, you are exhausted, whether from teaching, circle time, or activities.*

*(Anfal)* 

Another agreed, emphasizing the burden of overcrowded classrooms, which limits teachers’ capacity to manage time, resources, and meaningful interactions with children, noting:

*The large number of children in the classroom makes it difficult for teachers to provide enough materials or manage the class effectively. With 40 children, any activity with tools creates chaos.*

*(Merry)* 

Other teachers highlighted the limitations of transferring training into practice without systemic support. One teacher admitted:

*Honestly, I wish I could apply the knowledge I gained from the workshop, but I cannot unless I have the same program and same resources available to me in my school.*

*(Sara)* 

Veziroglu-Celik et al. (2025) commented in their study that a major difficulty to incorporate STEM teaching is the lack of access to appropriate resources. This highlights the dependency of successful implementation on institutional provision and structural support. However, the workshop resources were available on the website online which primarily supports her first language. The teacher, nonetheless, associated the availability of online resources with sensory materials in her school and expressed a desire to implement the workshop as it is within their curricular activities at the school. Interestingly, Tahani held an opposite view to Sara. She found that the workshop helped her to use the website link to design more activities that integrated STEM, explaining that:

*It is true that sometimes, as a teacher, I find it hard to apply STEM in our daily activities. But the trainer gave us a website that helped me to find more information and ideas about STEM. I used the idea of building a bridge with the children. We used recycled materials from our environment, such as wooden sticks, cups, aluminum, and colored adhesive tape, and we made King Fahad Bridge.*

*(Tahani)* 

This statement underscores the importance of the STEM website, to which teachers had full access and could draw on as many ideas as they wished to apply STEM in their classrooms. Her reflection shows how PD-supported digital resources can expand teachers’ repertoires, enabling them to adapt online ideas into meaningful, low-cost projects grounded in children’s local environment. Yara also identified the value of using local recycled materials to implement STEM in her daily classroom activities.

*I used a lot of recycled materials from our environment. I used a cardboard box, some batteries, some wires and a lamp to make an electrical circuit in our class. It was easy to do, and the children enjoyed trying it. Not only this, but I also tried to think first about what we already have in our environment that can be used and recycled in any activities.*

*(Yara)* 

Thus, using local materials and recycled resources from the environment helps to overcome many of the barriers that teachers face when implementing STEM in their classrooms, particularly those related to cost and limited access to commercial equipment. This finding is consistent with research showing that early year’s STEM can be effectively enacted through hands-on activities using every day or recycled materials, which both reduce resource constraints and foster creativity, problem-solving, and environmental responsibility among young children (Yabaş & Abanoz, 2024).

A further dimension of challenge was raised in relation to children’s unequal access to resources outside the classroom. As one teacher explained:

*Some children are very intelligent and creative, but at home they don’t have access to the environments and materials they need. We try to provide everything for them here in the kindergarten—papers, colors, whatever is available—but at home it is absent.*

*(Anfal)* 

This highlights how socio-economic disparities affect children’s opportunities to benefit equally from STEM learning. Finally, cultural expectations sometimes conflicted with STEM activities. One teacher shared:

*Sometimes we do water or measurement activities, like filling containers with sponges and recording the weights. This is also math. But some parents refuse, saying they don’t want their children to get dirty. Even when we ask parents to bring simple materials for activities, some do not agree and may even keep their child at home that day.*

*(Sara)* 

Such accounts illustrate how parental attitudes and lack of support created additional barriers, limiting children’s participation in STEM-based play. Together, these findings highlight that while professional development enhanced teachers’ readiness, structural conditions (large classes, lack of resources, time pressure), systemic gaps (inconsistent program provision), socio-economic disparities, and cultural constraints significantly hindered the translation of training into consistent practice. This aligns with research showing that sustainable STEM integration requires not only teacher training but also systemic reforms, resource provision, and community engagement (Leung, 2023; OECD, 2022).

## 5. Conclusions

The first objective for this research project was to analyse the impact of a targeted professional development on STEM education for early childhood teachers within the Saudi Arabian education context. The results indicate a statistically significant improvement in participants’ overall evaluations of STEM knowledge, conceptual understanding, and confidence after attending the workshop. Prior to the workshop, responses reflected an average level of understanding, whereas post-workshop evaluations demonstrated a high level of knowledge and confidence. The statistical analysis confirms that the professional development program had a substantial positive impact, enhancing early childhood teachers’ knowledge of STEM concepts as well as their self-confidence in applying them. The qualitative interviews clarified the professional development program significantly strengthened teachers’ capacity to integrate STEM meaningfully into early childhood settings, enriching literacy, numeracy, storytelling, and play-based learning.

The second objective identified how early childhood teachers apply their knowledge derived from the professional development program into practice when teaching STEM within the Saudi Arabian education context. This was evident when teachers applied workshop concepts in practical and creative ways, embedding STEM across daily routines, learning areas, and classroom activities, and making connections that were developmentally appropriate, engaging, and culturally responsive. The PD fostered a holistic understanding of STEM as interconnected rather than subject-specific, enabling teachers to support children’s curiosity, confidence, and creativity through hands-on, inquiry-driven experiences. It also extended teachers’ engagement beyond the classroom, prompting community-linked initiatives that made STEM visible to families and local institutions. At the same time, persistent systemic challenges—including large class sizes, time and resource constraints, socio-economic disparities, and varying levels of parental support limited consistent implementation. This highlights the need for broader structural and policy support. Overall, the study indicates that well-designed PD can transform early years educators’ beliefs and practices, but sustained impact depends on institutional resources, supportive school cultures, and partnerships that reinforce STEM-rich, play-based learning environments.

## 6. Limitations

One limitation of this study lies in its relatively short timeframe and reliance on self-reported data, which may not fully capture the long-term impact of the professional development program on teachers’ actual classroom practices. Although surveys and interviews provided valuable insights into participants’ attitudes and confidence, these methods are subject to response bias, as participants may have reported more positive outcomes due to social desirability. Moreover, the study did not include classroom observations or children’s learning outcomes, which could have offered a more comprehensive understanding of how professional learning translated into pedagogical change and improved STEM learning experiences for children.

Another limitation concerns the context-specific nature of the study, which restricts the generalizability of its findings beyond the Saudi Arabian early childhood education setting. The implementation of STEM education is shaped by cultural, institutional, and policy-related factors that may differ significantly in other regions or educational systems. Additionally, the sample size and diversity of participants may not have been sufficient to represent the full range of teachers’ experiences and challenges across the country. Future research would benefit from longitudinal studies involving classroom-based observations, broader participant representation, and the inclusion of school leadership perspectives to better understand the sustainability and scalability of STEM teaching practices in early childhood education.

## Figures and Tables

**Table 1:** Demographic Information of Qualitative Study Participants.

Participant	Years of Experience	School Type	City
Tahani	17	Public School	Madinah
Reem	7	Private School	Madinah
Abeer	9	International School	Madinah
Merry	15	Public School	Madinah
Qadian	21	Public School	Madinah
Banan	14	Public School	Madinah
Nadiyah	17	Private School	Madinah
Sara	9	Public School	Madinah
Aish	10	International School	Jeddah
Abrar	10	Public School	Jeddah
Yara	12	Private School	Jeddah
Noha	15	International School	Jeddah
Anfal	16	Public School	Jeddah
Areej	19	Private School	Jeddah
Nada	8	International School	Jeddah
Asmaa	10	Public School	Jeddah

**Table 2:** Correlation coefficients between the scores for each question in the survey and the total scores for the survey.

Items	Correlation Coefficient	*p*-Value
I need training to teach STEM.	0.79	0.01
I confidently incorporate STEM programs based on children’s interests.	0.82	0.01
I think STEM education is important equally for boys and girls.	0.75	0.01
Boys and girls equally benefit from STEM education.	0.83	0.01

**Table 3:** Reliability test results for the survey.

Axis	Items	Cronbach’s Alpha
The Survey	4	0.81

**Table 4:** Significance of the differences between the mean scores of the research sample’s responses regarding ‘I need training to teach STEM to the children’ before and after attending the workshop.

Item	Workshop Attendance	Mean	SD	Level	T	DF	*p*-Value
I need training to teach STEM to the children.	Before	3.14	1.13	Medium	8.64	76	0.001
	After	4.51	0.70	High			
I confidently incorporate STEM programs based on children’s interests.	Before	2.58	1.40	Low	8.96	76	0.001
	After	4.21	0.75	High			
I think STEM education is important equally for boys and girls.	Before	3.18	1.28	Medium	7.78	76	0.001
	After	4.55	0.62	High			
Boys and girls equally benefit from STEM education.	Before	3.26	0.94	Medium	12.05	76	0.001
	After	4.68	0.55	High			
Overall evaluation.	Before	3.04	0.67	Average	15.46	76	0.001
	After	4.48	0.39	High			

**Table 5:** Summary of Qualitative Themes and Representative Teacher Excerpts.

Theme	Analytic Focus (What This Theme Captures)	Representative Excerpt
Theme 1: Applying Workshop Knowledge in Practice	This theme captures how teachers translated professional development content into concrete classroom practices, including the direct use of workshop strategies, instructional innovation, and changes in pedagogical thinking.	*“So, I tried it and brought it to class; from it, we made a rocket, and the children truly invented things.”*
Theme 2: Holistic Integration of STEM in Play-Based Learning	This theme reflects teachers’ use of play-based, creative, and integrated STEM approaches, where engineering, creativity, and thematic learning were embedded naturally within children’s play experiences.	*“It encourages thinking and creativity, making them wonder how it worked and how it moved… even we adults enjoyed it.”*
Theme 3: Making STEM Visible: Community Links, Equity, and Engagement	This theme highlights how teachers connected STEM learning to children’s social worlds, values, and broader engagement, including collaboration, inclusion, and fostering equitable participation in STEM activities.	*“We teach them how to work together, how to care for each other, and how to cooperate.”*
Theme 4: Challenges in Translating STEM Professional Development into Practice	This theme captures the practical, contextual, and structural challenges teachers faced when implementing STEM, including classroom constraints, resource limitations, and the need to adapt activities to learners’ differences.	*“Sometimes the ideas are good, but applying them in class needs more support and adjustment for the children.”*

## Data Availability

The data presented in this study are available on request from the corresponding author due to ethical responsibilities to protect participant confidentiality and privacy.

## References

[B1-behavsci-16-00127] Akcay Malcok B., Ceylan R. (2022). The effects of STEM activities on the problem-solving skills of 6-year-old preschool children. European Early Childhood Education Research Journal.

[B2-behavsci-16-00127] Aldemir J., Kermani H. (2017). Integrated STEM curriculum: Improving educational outcomes for head start children. Early Child Development & Care.

[B3-behavsci-16-00127] Alexander C., Knezek G., Christensen R., Tyler-Wood T., Bull G. (2014). The impact of project-based learning on pre-service teachers’ technology attitudes and skills. Journal of Computers in Mathematics & Science Teaching.

[B4-behavsci-16-00127] Alghamdi A. (2022). Exploring early childhood teachers’ beliefs about STEAM education in Saudi Arabia. Early Childhood Education.

[B5-behavsci-16-00127] Alghamdi A. (2023). Culture in early childhood education: Insights into Saudi preschool teaching. Journal of Education and Learning (EduLearn).

[B6-behavsci-16-00127] Alharthi M., Aljohani A. (2025). Unveiling the magic of play: An exploration of pre-service teachers’ understandings of child-led play and unstructured play in Saudi Arabian preschools. Journal of Early Childhood Teacher Education.

[B7-behavsci-16-00127] Almalki A. (2023). Cultural and religious perspectives on education of Saudi Arabia. Journal of Science, Technology and Innovation Policy.

[B8-behavsci-16-00127] Australian Education Department (2021). Introductory material—What is STEM?.

[B9-behavsci-16-00127] Ba Akhlagh S., Allehyani H. S., Yang W., Kewalramani S., Senthil J. (2023). Saudi early childhood female teachers’ perceptions of children’s gender stereotypes when implementing STEM. Science, technology, engineering, arts, and mathematics (STEAM) education in the early years; achieving the sustainable development goals.

[B10-behavsci-16-00127] Bagiati A., Evangelou D. (2015). Engineering curriculum in the preschool classroom: The teacher’s experience. European Early Childhood Education Research Journal.

[B11-behavsci-16-00127] Bates C. C., Morgan D. N. (2018). Seven elements of effective professional development. The Reading Teacher.

[B12-behavsci-16-00127] Boice K. L., Jackson J. J., Alemdar M., Rao A. E., Grossman S., Usselam M. (2021). Supporting teachers on their STEAM journey: A collaborative STEAM teacher training program. Education Sciences.

[B13-behavsci-16-00127] Boz T. (2023). Teacher professional development for STEM integration in elementary/primary schools: A systematic review. International Electronic Journal of Elementary Education.

[B14-behavsci-16-00127] Braun V., Clarke V. (2021). Thematic analysis: A practical guide.

[B15-behavsci-16-00127] Chesloff J. D. (2013). STEM education must start in early childhood. Education Week.

[B16-behavsci-16-00127] Cohrssen C., Garvis S. (2022). Embedding steam in early childhood education and care.

[B17-behavsci-16-00127] Copur-Gencturk Y., Thacker I. (2020). A comparison of perceived and observed learning from professional development: Relationships among self-reports, direct assessments, and teacher characteristics. Journal of Teacher Education.

[B18-behavsci-16-00127] Creswell J. W., Clark V. L. (2017). Designing and conducting mixed methods research.

[B19-behavsci-16-00127] Darling-Hammond L., Hyler M. E., Gardner M. (2017). Effective teacher professional development.

[B20-behavsci-16-00127] Davila Dos Santos E., Albahari A., Díaz S., De Freitas E. C. (2021). ‘Science and technology as feminine’: Raising awareness about and reducing the gender gap in STEM careers. Journal of Gender Studies.

[B21-behavsci-16-00127] Deehan J., Redshaw S., Danaia L., Postlethwaite F., Donnelly A., Morris C. (2025). Understanding STEM beyond the cities: A comprehensive review of non-metropolitan STEM education research. International Journal of Educational Research Open.

[B22-behavsci-16-00127] DeJarnette N. K. (2018). Implementing STEAM in the early childhood classroom. European Journal of STEM Education.

[B23-behavsci-16-00127] Fetters M. D., Curry L. A., Creswell J. W. (2013). Achieving integration in mixed methods designs—Principles and practices. Health Services Research.

[B24-behavsci-16-00127] Field A. (2018). Discovering statistics using IBM SPSS Statistics.

[B25-behavsci-16-00127] Ghazali A., Ashari Z. M., Hardman J. (2024). A scoping review on STEM education: The best practices recorded through previous studies in early childhood education setting. International Journal of Education in Mathematics, Science and Technology.

[B26-behavsci-16-00127] Haney J. J., Lumpe A. T., Czerniak C. M., Egan V. (2002). From beliefs to actions: The beliefs and actions of teachers implementing change. Journal of Science Teacher Education.

[B27-behavsci-16-00127] IBM Corp (2022). IBM SPSS statistics for windows *(Version 29.0) [Computer software]*.

[B28-behavsci-16-00127] Ji L., Aziku M., Qiang F., Bin Z. (2024). Leveraging professional learning communities in linking digital professional devel-opment and instructional integration: Evidence from 16,072 STEM teachers. International Journal of STEM Education.

[B29-behavsci-16-00127] Johnson C. C. (2013). Conceptualizing integrated STEM education. School Science and Mathematics.

[B30-behavsci-16-00127] Keene T., Garvis S. (2024). STEM in Australian early childhood education. Journal of Information Processing.

[B31-behavsci-16-00127] Knaus M. (2023). Growing minds—The importance of STEM in early childhood.

[B32-behavsci-16-00127] Leung K. C. (2023). Shifting pedagogies in early childhood STEM: Challenges and strategies in resource-constrained contexts. Education Sciences.

[B33-behavsci-16-00127] MacDonald A., Danaia L., Sikder S., Huser C. (2021). Early childhood educators’ beliefs and confidence regarding STEM education. International Journal Early Childhood.

[B34-behavsci-16-00127] Madani R. A. (2020). Teaching challenges and perceptions on STEM implementation for schools in Saudi Arabia. European Journal of STEM Education.

[B35-behavsci-16-00127] Mohamad Hasim S., Rosli R., Halim L., Capraro M. M., Capraro R. M. (2022). STEM professional development activities and their impact on teacher knowledge and instructional practices. Mathematics.

[B36-behavsci-16-00127] OECD (2022). Education at a glance 2022: OECD indicators.

[B37-behavsci-16-00127] Ong E. T., Ayob A., Ibrahim M. N., Adnan M., Shariff J., Ishak N. (2016). The effectiveness of an in-service training of early childhood teachers on STEM integration through project-based inquiry learning (PIL). Journal of Turkish Science Education (TUSED).

[B38-behavsci-16-00127] Park M. H., Dimitrov D. M., Patterson L. G., Park D.-Y. (2017). Early childhood teachers’ beliefs about readiness for teaching science, technology, engineering, and mathematics. Journal of Early Childhood Research.

[B39-behavsci-16-00127] Patton M. Q. (2015). Qualitative research & evaluation methods.

[B40-behavsci-16-00127] Penuel W. R., Fishman B. J., Yamaguchi R. (2007). What makes professional development effective? Strategies that foster curriculum implementation. American Educational Research Journal.

[B41-behavsci-16-00127] Revák I. M., Csernoch M., Czimre K., Dávid Á., Tóth B. K., Malmos E., Suto E., Kurucz D. (2024). A systematic review of STEM teaching-learning methods and activities in early childhood. Eurasia Journal of Mathematics, Science and Technology Education.

[B42-behavsci-16-00127] Ritoša A., Åström F., Björck E., Borglund L., Karlsson E., McHugh E., Nylander E. (2023). Measuring children’s engagement in early childhood education and care settings: A scoping literature review. Educational Psychology Review.

[B43-behavsci-16-00127] Roberts P., Knaus M. (2018). STEM in early childhood education: Using the inquiry approach to scaffold learning. New Zealand International Research in Early Childhood Education.

[B44-behavsci-16-00127] Slavit D., Nelson T. H., Lesseig K. (2016). The teachers’ role in developing, opening, and nurturing an inclusive STEM-focused school. International Journal of STEM Education.

[B45-behavsci-16-00127] Stone B. (2024). Science, technology, engineering, art, and mathematics: STEAM the impact of authentic early childhood STEM experiences on cognitive development. International Journal of the Whole Child.

[B46-behavsci-16-00127] Sylva K., Melhuish E., Sammons P., Siraj-Blatchford I., Taggart B. (2010). Early childhood matters: Evidence from the effective pre-school and primary education project.

[B47-behavsci-16-00127] Thibaut L., Ceuppens S., De Loof H., Van De Keere K., Verschaffel L., Depaepe F. (2018). Integrated STEM education: A systematic review of instructional practices in secondary education. European Journal of STEM Education.

[B48-behavsci-16-00127] Tytler R., Anderson J., Li Y. (2020). STEM education for the twenty-first century. Integrated approaches to STEM education. Advances in STEM education.

[B49-behavsci-16-00127] UNESCO (2019). Early childhood care and education.

[B50-behavsci-16-00127] Veziroglu-Celik M., Ozkaya S., Kacar G., Senturk Z. (2025). STEAM in early childhood: An analysis towards teachers’ and children’s perspectives. Early Childhood Education.

[B51-behavsci-16-00127] Victoria Department of Education and Training (2017). Making the most of childhood: The importance of early years.

[B52-behavsci-16-00127] Vision 2030 (2016). Kingdom of Saudi Arabia.

[B53-behavsci-16-00127] Vygotsky L. S. (1978). Mind in society: The development of higher psychological processes.

[B54-behavsci-16-00127] Walshe P., Commins A., McDonnell C., Kelly B. (2025). ‘STEAM from the Start’: Proposing a conceptual framework for the development and implementation of a STEAM training intervention for early childhood educators. European Journal of STEM Education.

[B55-behavsci-16-00127] Wan Z. H., Jiang Y., Zhan Y. (2020). STEM education in early childhood: A review of empirical studies. Early Education and Development.

[B56-behavsci-16-00127] World Health Organization (2020). Improving early childhood development: WHO guideline.

[B57-behavsci-16-00127] Yabaş D., Abanoz T. (2024). Integrated STEM teaching: Innovative STEM training for preschool and primary school teachers. Journal of Education and Future.

[B58-behavsci-16-00127] Zendler A., Seitz C., Klaudt D. (2018). Instructional methods in STEM education: A Cross-contextual study. EURASIA Journal of Mathematics, Science and Technology Education.

